# The PLSR-ML fusion strategy for high-accuracy leaf potassium inversion in karst region of Southwest China

**DOI:** 10.3389/fpls.2025.1620971

**Published:** 2025-07-07

**Authors:** Zhihao Song, Wen He, Yuefeng Yao, Ling Yu, Jinjun Huang, Yong Xu, Haoyu Wang

**Affiliations:** ^1^ College of Geomatics and Geoinformation, Guilin University of Technology, Guilin, China; ^2^ Guangxi Key Laboratory of Plant Conservation and Restoration Ecology in Karst Terrain, Guangxi Institute of Botany, Guangxi Zhuang Autonomous Region and Chinese Academy of Sciences, Guilin, China; ^3^ School of Computer Science and Engineering, Guilin University of Aerospace Technology, Guilin, China

**Keywords:** karst region, leaf potassium content, machine learning, fractional differentiation, spectral reflectance

## Abstract

Potassium is a critical macronutrient for plant growth, yet accurately and rapidly estimating its content in karst regions remains challenging due to complex terrestrial conditions. To address this, we collected leaf potassium content and reflectance data from 301 plant samples across nine karst regions in Guangxi Province. Our results showed that hybrid models combining Partial Least Squares Regression (PLSR) with three machine learning algorithms—Random Forest (RF), Extreme Gradient Boosting (XGBoost), and Multi-Layer Perceptron (MLP)—namely PLSR-RF, PLSR-XGBoost, and PLSR-MLP, demonstrated exceptional accuracy in estimating leaf potassium content. Validation coefficient of determination (R²) values reached 0.89, 0.94, and 0.96, respectively—representing improvements of 206%, 147%, and 108% over standalone algorithms. This performance gain was attributed to rigorous overfitting control: PLSR’s dimensionality reduction synergized with ensemble machine learning (RF, XGBoost, MLP) to eliminate redundant spectral features while retaining predictive signals. Furthermore, fractional differentiation preprocessing significantly improved the correlation between spectral reflectance and potassium content, enhancing model robustness. Two spectral regions (700–1100 nm, 1400–1800 nm) were identified as key predictors, aligning with known potassium-related biochemical absorption features. Collectively, the integration of these strategies offers a robust framework for nutrient monitoring in ecologically fragile karst ecosystems.

## 1 Introduction

The karst landscapes of southwestern China constitute a globally significant geomorphological system (D’Ettorre et al., 2024). Characterized by distinctive lithological structures and heterogeneous vegetation assemblages, this ecologically fragile region serves as a vital reservoir of terrestrial biodiversity. Nevertheless, intensive anthropogenic activities—particularly shifting slash-and-burn agriculture and unsustainable slope farming practices—have induced substantial degradation of surface vegetation cover (Jiang et al., 2014). This degradation may adversely affect the availability of essential nutrient such as potassium, which plays a critical role in plant growth by regulating water balance, facilitating nutrient transport (Wang et al., 2013; Hasanuzzaman et al., 2018), and enhances plant resilience against biotic and abiotic stressors (Anschütz et al., 2014). Foliar potassium concentration serves as a robust phytochemical indicator strongly correlated with plant physiological status, providing critical insights into vegetation health assessment. Consequently, precise quantification of foliar potassium levels emerges as a methodological imperative for elucidating plant adaptive strategies in degraded karst ecosystems.

Conventional laboratory methods for leaf potassium analysis, relying on destructive wet chemistry techniques, face inherent limitations in operational efficiency and scalability. In contrast, hyperspectral reflectance technology has emerged as a transformative non-destructive solution, enabling rapid *in situ* nutrient assessment through advanced spectroscopic platforms. The integration of hyperspectral remote sensing systems has particularly enhanced real-time potassium monitoring capabilities, demonstrating remarkable success in precision agriculture applications (Lin et al., 2024; Azadnia et al., 2023). However, species-specific variations result in differing spectral band sensitivities to potassium content across plant taxa. Current research has yet to fully resolve uncertainties in characterizing potassium-related spectral responses, necessitating further investigation into their underlying mechanisms. For example, Lu et al. (2020) found that the spectral reflectance of rice leaves in the shortwave infrared region (1300–2000 nm) is particularly sensitive to potassium content. Similarly, Lyu et al. (2023) identified potassium-sensitive bands in grape leaves at 410 nm, 490–500 nm, and 1242 nm. These discrepancies between rice and grape studies underscore the variability in potassium-sensitive spectral regions across species, highlighting both the challenges in universal band selection and the critical need for taxa-specific calibration. This variability becomes particularly relevant in ecologically unique regions such as karst landscapes, which host specialized plant communities. Karst-adapted species exhibit distinct spectral signatures compared to non-karst flora due to their divergent evolutionary adaptations and environmental stressors (Yue et al., 2010). Consequently, region-specific studies are imperative to map the spectral sensitivity patterns of leaf potassium in karst ecosystems, enabling accurate nutrient monitoring and supporting ecological conservation in these biodiverse yet fragile habitats.

While spectral information enables precise characterization of potassium signatures in leaf spectral response curves, noise interference remains a significant concern (Xie et al., 2020). Hyperspectral data acquisition is inherently susceptible to artifacts introduced by sample properties (e.g., particle size and surface texture) and environmental variability (Kong et al., 2023). Spectral differentiation transformations serve as a robust preprocessing technique to mitigate background noise and unwanted spectral reflectance variations. These transformations enhance spectral sensitivity, amplify diagnostic features, and optimize predictive model performance (Yang C, et al., 2021). First- and second-order derivatives are widely employed to improve spectral signal-to-noise ratios. However, integer-order differentiation exhibits limitations in resolving subtle spectral features when curvature variations are gradual, often leading to feature loss (Li et al., 2024). In contrast, fractional differentiation operates at finer computational intervals, enabling enhanced spectral information extraction from *in situ* leaf measurements (Benkhettou et al., 2015). In addition, using fractional differentiation can further sharpen peak shapes and perform better in detecting subtle signal changes in positive and negative spectral peaks (Tan et al., 2024). This methodological refinement directly translates to improved precision in estimating critical biochemical parameters, such as foliar potassium levels, which will be rigorously evaluated in our experimental framework.

In the field of nutrient content inversion, mainstream empirical approaches can be broadly categorized into four types (Berger et al., 2020): empirical parameter regression (Jay et al., 2017), linear nonparametric regression (Furlanetto et al., 2024), physically based methods, and nonlinear nonparametric regression (i.e., machine learning) (Furlanetto et al., 2023; Flynn et al., 2023). Empirical parameter regression typically employs narrowband vegetation indices (e.g., NIR/SWIR combinations) for rapid estimation of nutrients. However, due to the lack of distinct absorption features for certain elements and the influence of spectral signal coupling, these methods often suffer from limited generalizability (Li et al., 2021). Linear nonparametric regression techniques such as Partial Least Squares Regression (PLSR) and Principal Component Regression (PCR) utilize full-spectrum information and avoid manual feature selection. Still, their reliance on linear assumptions makes it difficult to capture the complex nonlinear relationships between spectral responses and plant biochemical properties (Atzberger et al., 2010). Physically based radiative transfer models (e.g., PROSAIL) aim to simulate the nutrient–spectrum relationship from a mechanistic perspective. Nonetheless, the weak absorption features of nutrients can be easily confounded with canopy water content and structural parameters, leading to ill-posed inversion problems (Féret et al., 2019). In contrast, machine learning methods are well-suited for nutrient estimation due to their strong capabilities in modeling complex nonlinear relationships and handling large-scale datasets (He et al., 2021).

However, significant challenges in model fitting persist when applying machine learning algorithms to vegetation parameter estimation (Doktor et al., 2014). The performance of machine learning models critically depends on feature selection - excessively large feature sets or overcomplicated architectures frequently lead to overfitting, compromising both training accuracy and model generalizability. Conversely, insufficient feature quantities and oversimplified models may result in underfitting. This issue is particularly pronounced in field spectroscopy data characterized by high dimensionality and multicollinearity. To address these challenges, researchers have implemented multiple mitigation strategies: (1) expanding training datasets to improve statistical representation; (2) employing dimensionality reduction techniques; (3) adopting robust cross-validation protocols; (4) applying regularization methods (Zhang et al., 2021); and (5) developing ensemble learning frameworks (Wang R, et al., 2020).

Hyperspectral data is characterized by high dimensionality and multivariate features, and the issue of feature redundancy has yet to be effectively resolved (Liu et al., 2021). This necessitates systematic dimensionality reduction of hyperspectral data to ensure model robustness. Notable implementations include Cao et al. (2021), who successfully mitigated overfitting in maize leaf nitrogen estimation through optimized spectral compression, and Ni et al. (2024) achieving superior predictive performance (R²=0.98) in sucrose quantification models via principal component analysis (PCA). This empirical evidence collectively substantiates that dimensionality reduction techniques, particularly PCA, significantly enhance both model accuracy (p<0.01) and algorithmic stability compared to untreated hyperspectral inputs. Building upon these methodological advancements, our study innovatively integrates partial least squares (PLS)-optimized PCA with ensemble machine learning frameworks to establish a robust estimation model for leaf potassium content in karst ecosystems, specifically designed to improve generalizability across heterogeneous geological environments.

Based on field spectrometer data, this study used a fractional differential spectroscopy method combined with multiple models to estimate the potassium content in the leaves of mixed forests in the Guangxi karst region. The main objectives of this research are as follows: (1) To assess the distribution of wavelengths sensitive to potassium content in plant leaves in the karst region; (2) To explore the role of fractional differentiation in estimating potassium content in karst plant leaves based on spectroradiometer data; and (3) To investigate whether combined models can overcome the overfitting issues encountered in machine learning models when estimating potassium content in karst plant leaves.

## 2 Materials and methods

### 2.1 Study area

**Figure 1:**
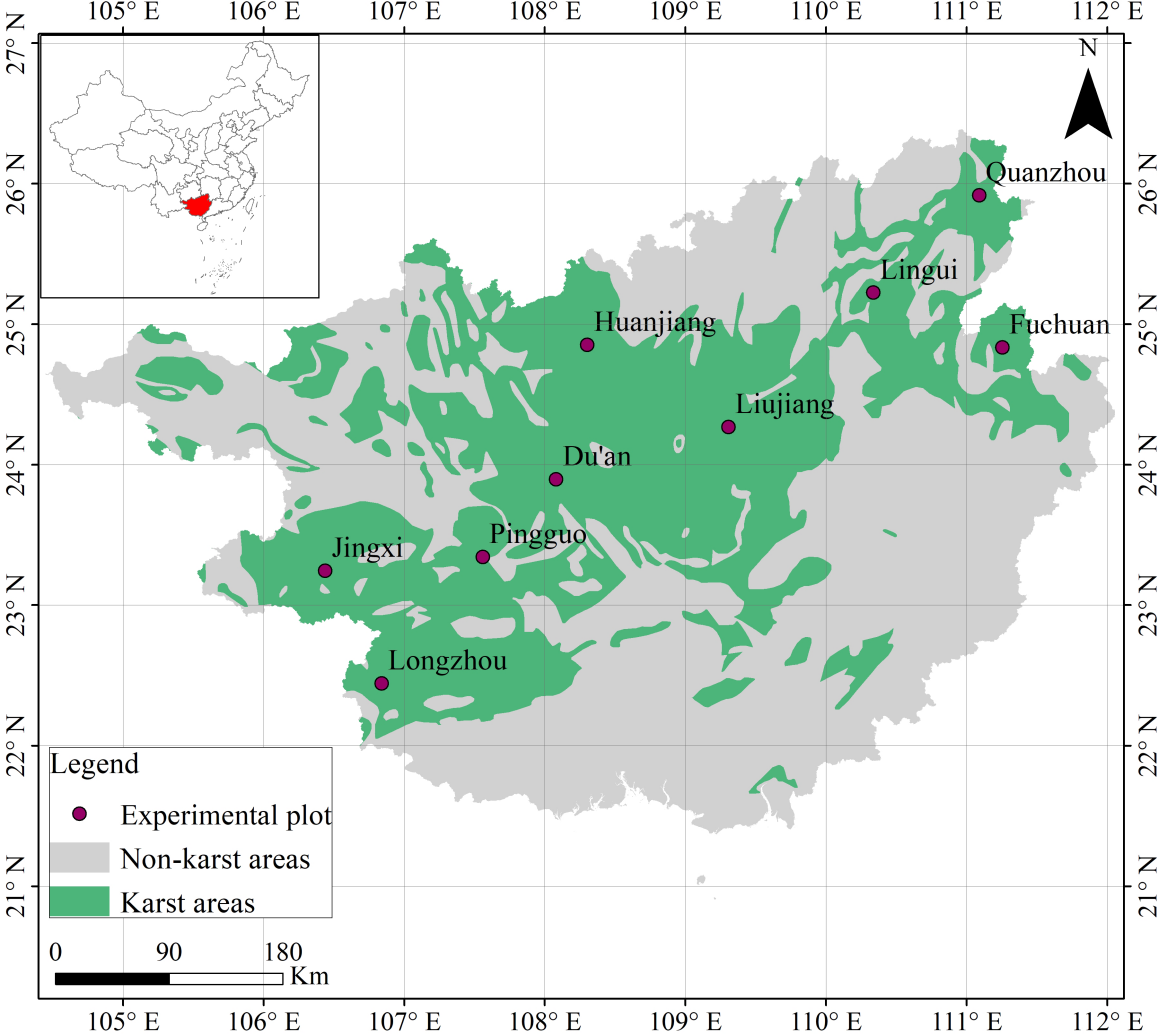
Location of the nine sample plots.

The investigation was conducted in the karst-dominated terrain of Guangxi Zhuang Autonomous Region, Southwest China (20°54′-26°24′N, 104°28′-112°04′E; **Figure 1**). This geomorphologically complex area exhibits altitudinal gradients ranging from coastal plains (0 m) to montane systems (2141 m ASL), bisected by the Tropic of Cancer and bounded by tropical marine systems to the south. These latitudinal and topographic configurations engender a monsoonal climate regime with pronounced seasonality, manifesting in mean annual temperatures of 17.5-23.5°C and precipitation gradients from 841.2 mm (leeward basins) to 3387.5 mm (windward slopes). Nine standardized plots (200 m² each) were established across karst terrains, covering three vegetation succession stages: primary forests, secondary forests, and shrublands. This stratified design effectively captures karst ecosystem heterogeneity.

### 2.2 Data collection

Longitudinal foliar sampling spanned July 2018 to September 2020 across all study plots. Within each plot, phyllosphere specimens were systematically collected from 8–15 dominant species, establishing a comprehensive karst flora spectral database comprising 301 samples representing 37 families, 59 genera, and 70 species. To ensure spatial representativeness, sampling followed triaxial orientation protocols (0°[N], 120°, and 240°) within the horizontal plane.

Spectral acquisition employed a high-resolution field spectroradiometer (Fieldspec4, ASD Inc., USA) with 3 nm VNIR (350–1000 nm) and 8 nm SWIR (1001–2500 nm) spectral resolution (Shah et al., 2019). Three photometric replicates per tree were obtained through standardized protocol: 1) periodic radiometric calibration (10-minute intervals) using integrated reference panels; 2) constrained by field operation limitations (4-hour battery endurance), two mature leaves per branch underwent non-destructive scanning; 3) branch-level spectral signatures were averaged to derive tree-specific reflectance profiles.

Post-spectral analysis, target leaves were immediately preserved in sterile bags (Whirl-Pak^®^) under controlled conditions (ICERSICE940 incubator, 4°C). Samples underwent laboratory processing within 24 h: 1) oven-drying at 75°C to constant mass; 2) mechanical homogenization to 100-mesh particle size; 3) quantitative potassium determination via flame photometric analysis (Sherwood 410, ± 0.01 ppm detection limit) following standard digestion protocols (Reddy and Veeranki, 2013).

### 2.3 Methodology

#### 2.3.1 Fractional differentiation

The fractional differentiation extends the concept of traditional integer-order differentiation to any arbitrary order, enabling continuous interpolation between integer orders (Hong et al., 2019). This method emphasizes subtle changes in spectral information (Wang Z, et al., 2020). Currently, the classic definitions of fractional differentiation include Riemann-Liouville (R-L), Grünwald-Letnikov (G-L), and Caputo (Pu et al., 2008; Wang et al., 2018). In this study, the Grünwald-Letnikov (G-L) definition was mainly adopted to derive the differentiation to the n-th order, as shown in Equation 1.


(1) $$d^{v}f(x)=\underset{h\to \infty }{lim}\frac{1}{h^{v}}\sum_{m=0}^{\frac{t-a}{h}}{\left(-1\right)}^{m}\frac{\Gamma (v+1)}{m!\Gamma (v-m+1)}f(x-mh)$$

where v is the order of differentiation, h is the step size, t and a are the upper and lower bounds of differentiation, respectively, and Γ(⋅) is the Gamma function, as defined in Equation 2.


(2) $$\text{Γ}(\beta )=\int_{0}^{\infty }e^{-t}t^{\beta -1}dt=(\beta -1)!$$

where *β* is an arbitrary variable. In this study, the leaf spectra were differentiated within the range of 0 to 3 orders (at intervals of 0.1 order).

#### 2.3.2 Partial least squares regression

Partial Least Squares Regression (PLSR) is a multivariate data analysis technique that combines the features of Principal Component Analysis (PCA) and Multiple Linear Regression (MLR). It is used to predict a set of dependent variables from a large number of collinear independent variables. This method assumes that the datasets of independent and dependent variables are $Z={\left[z_{1},z_{2},\cdots z_{k}\right]}_{n\times k}$ and $Q={\left[q\right]}_{n\times 1}$ , respectively. First, the first latent variable *f_1_* ​ is extracted from Z, which is a linear combination of $z_{1},z_{2},\cdots z_{k}$ , and maximizes the correlation with the dependent variable *Q*. Then, a regression model is established for *Q* using *f_1_* ​​. If the regression equation achieves the desired accuracy, component extraction is stopped; otherwise, the next component is extracted until the model reaches a satisfactory level of accuracy. The regression model is given by Equation 3, and each latent variable is defined as shown in Equation 4:


(3) $$q=f_{1}a_{1}+f_{2}a_{2}+\cdots +f_{k}a_{k}$$

(4) $$f_{m}=w_{m1}z_{1}+w_{m2}z_{2}+\cdots +w_{mk}z_{k}$$

where *m* is the number of principal components, *k* is the number of independent variables, *a* is the regression coefficient of *y* with respect to *f*, and *w* is the linear coefficient of *f* with respect to *z*.

#### 2.3.3 Random forest

Random Forest (RF) is a machine learning algorithm based on decision trees (Breiman, 2001). RF resamples multiple samples from the training dataset and constructs a decision tree for each sample. Finally, the output value is calculated as the average of the predictions from all decision trees (Yang T, et al., 2021). RF has two important parameters: the number of trees and the number of features considered for splitting at each node. Initially, the number of decision trees was set to 50, and was then gradually increased in steps of 50 until it reached 200. The feature parameters for each node split were set as sqrt, log2, and 10. The optimal parameters were determined through grid search (Yang and Shami, 2020a).

#### 2.3.4 Extreme gradient boosting

XGBoost is an improved algorithm based on Gradient Boosted Decision Trees (GBDT), proposed by Chen and Guestrin (2016), which efficiently constructs boosted trees and supports parallel computation. Compared with traditional GBDT, which only utilizes first-order differential information, XGBoost performs a second-order Taylor expansion on the loss function, thereby improving the efficiency of finding the optimal solution. Finally, XGBoost obtains the overall prediction by summing the predictions of multiple decision trees, as shown in Equation 5.


(5) $${\hat{y}}_{n}=\sum_{n}^{M}f_{m}x_{n},f_{m}\in F$$

where ${\hat{y}}_{n}$ represents the final predicted value of the model, *M* denotes the number of combined decision trees, which is the number of trees to be tuned, $f_{m}$ is the *m*-th tree, $x_{n}$ represents the *n*-th input sample, and *F* is the set of all tree models.

#### 2.3.5 Multilayer perceptron

A Multilayer Perceptron (MLP) is a feedforward neural network composed of multiple neurons or nodes, which learns complex nonlinear mappings through connections between input and output vectors. It utilizes a parallel hierarchical structure consisting of an input layer, hidden layers, and an output layer, with information being transmitted through connection weights among these layers to predict target variables (Ehteram et al., 2020). In an MLP, the sum of the input signals received by a node is transformed through a nonlinear activation function to generate the output signal (Gardner and Dorling, 1998).


(6) $$s_{j}=\sum_{i=1}^{n_{0}}w_{ij}x_{i}+a_{j}$$

(7) $$z_{j}=f(s_{j})={\left(1+e^{-c_{j}}\right)}^{-1}$$

In Equations 6, 7, $s_{j}$ ​ represents the input to the *j*-th neuron in the hidden layer, ​ $a_{j}$ is the bias for the *j*-th neuron in the hidden layer, $w_{ij}$ is the weight between the *i*-th input neuron and the *j*-th neuron in the hidden layer, $f(b_{j})$ is the activation function, and $z_{j}$ is the output of the *j*-th neuron. The final output of the MLP is obtained by computing a weighted sum of the hidden layer outputs, as shown in Equation 8:


(8) $$O_{k}=\sum_{j=1}^{n_{1}}w_{jk}z_{j}+a_{k}$$

where $O_{k}$ ​ is the output of the *k*-th neuron in the output layer, $w_{jk}$ ​ is the weight between the *j*-th neuron in the hidden layer and the *k*-th neuron in the output layer, and *n_1​_* represents the number of neurons in the hidden layer.

#### 2.3.6 Combined models, sample segmentation, and accuracy assessment

The partial least squares regression (PLSR)-derived latent variables served as input variables for three machine learning architectures: RF, XGBoost, and MLP. Subsequently, the integrated models PLSR-RF, PLSR-XGBoost, and PLSR-MLP were established. This hybrid dimensionality reduction approach effectively mitigated high-dimensionality challenges inherent in spectral data while controlling algorithmic complexity. During latent variable extraction from fractionally differentiated spectra, we implemented a variance retention threshold, where the process was terminated once the cumulative explained variance reached 75%, to preserve critical spectral features.

To effectively split the data into training and validation sets, the train_test_split function from the scikit-learn library in Python 3.10 was used. This function allows for random splitting of the dataset into different subsets, ensuring the independence of model training and validation. The training set accounted for 4/5 of the total samples, while the validation set accounted for 1/5. The model accuracy was evaluated using the coefficient of determination (R²), mean squared error (MSE), and mean absolute error (MAE).

#### 2.3.7 Model parameter optimization

To ensure optimal predictive performance, the key hyperparameters of each model were systematically optimized. For the Partial Least Squares Regression (PLSR) model, the optimal number of components (n_components) was determined through exhaustive manual search over a predefined range (1 to 20) with model performance evaluated via 10-fold cross-validation. For the three machine learning models integrated with PLSR-Random Forest (RF), Extreme Gradient Boosting (XGBoost), and Multi-Layer Perceptron (MLP)—hyperparameter tuning was performed using grid search with 10-fold cross-validation (Yang and Shami, 2020a).

In the RF model, the primary parameters optimized included the number of trees (n_estimators, e.g., 100, 200, 300) and the maximum tree depth (max_depth, e.g., 5, 10, 15). For the XGBoost model, key parameters such as the learning rate (learning_rate, e.g., 0.01, 0.05, 0.1), maximum depth (max_depth), and the number of estimators (n_estimators) were adjusted. In the MLP model, optimization focused on the architecture of hidden layers (hidden_layer_sizes, e.g., (100), or (100, 50)), activation function (activation, e.g., ReLU), solver algorithm (solver, e.g., Adam), and the L2 regularization term (alpha).

## 3 Results

### 3.1 Descriptive statistics of the samples

**Figure 2:**
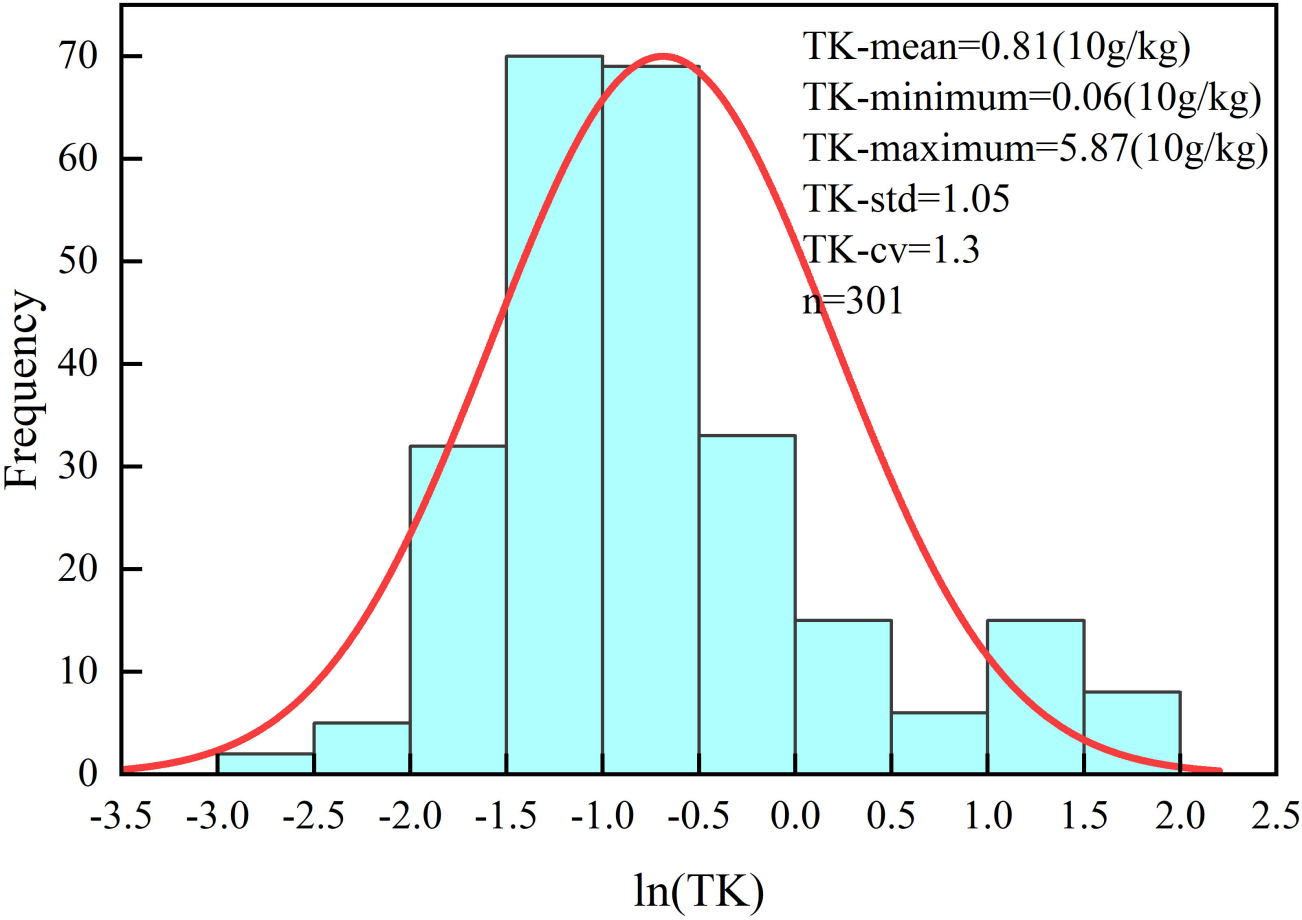
The leaf potassium content frequency distribution.

A total of 301 leaf samples were collected and analyzed for their total potassium content (expressed in units of 10 g/kg). The results showed that the potassium content ranged from 0.06 to 5.87, with a mean value of 0.81 (**Figure 2**). The coefficient of variation was calculated to be 1.30, indicating a high degree of variability among the samples. This substantial variation provides a solid foundation for model development and accuracy evaluation in subsequent analysis.

### 3.2 Fractional differentiation of reflectance and its correlation

**Figure 3:**
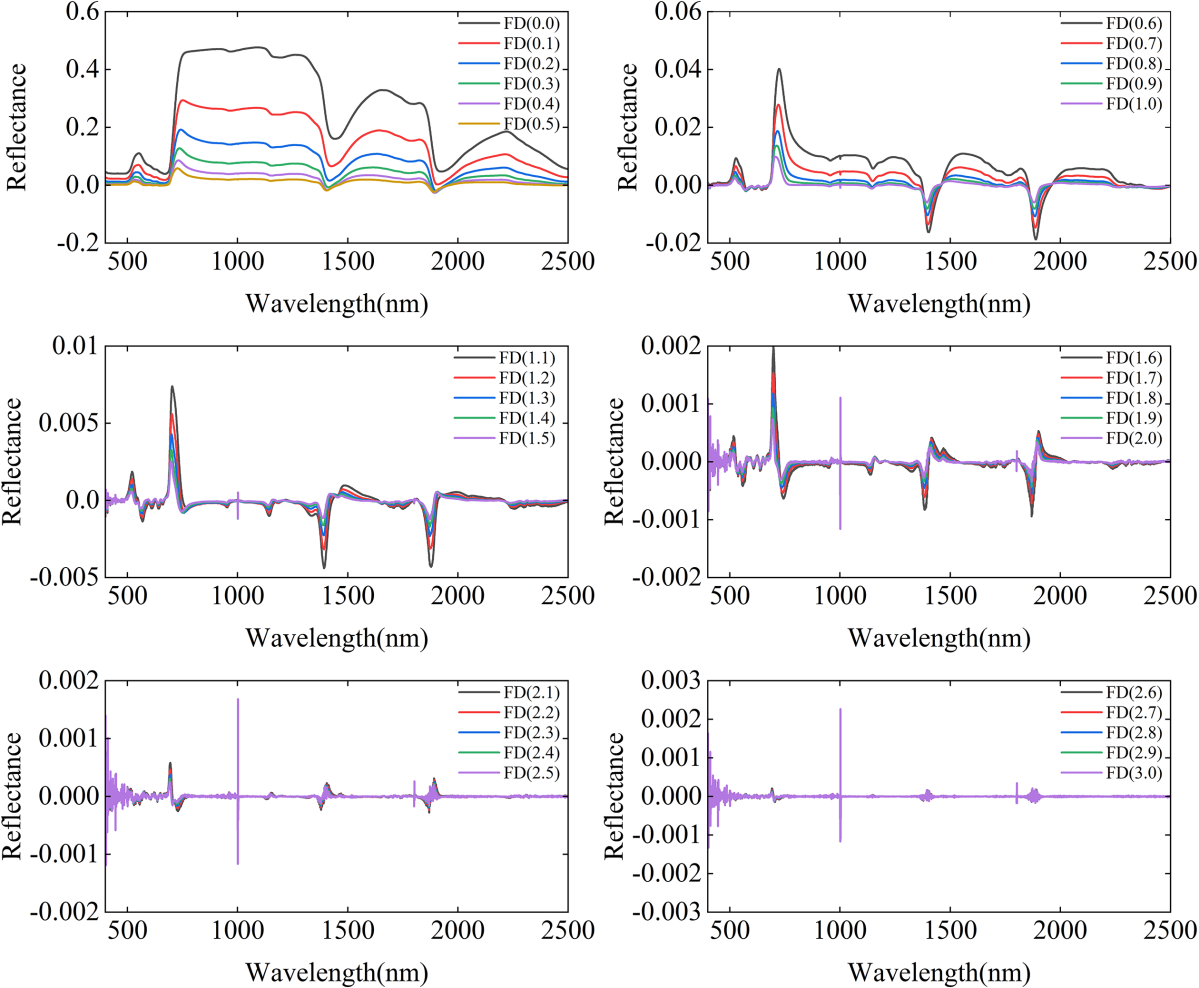
Effect of fractional differentiation orders from FD (0.0) to FD (3.0) on vegetation spectral reflectance: average reflectance spectra for each order.

**Figure 3** illustrates the variations in spectral reflectance with different fractional differentiations. Compared to integer-order differentiations (0th, 1st, 2nd, and 3rd), fractional differentiation exhibits smaller amplitudes and smoother transitions. This gradual transformation maintains the detailed features of the spectral curves and prevents the abrupt fluctuations typically observed in integer-order differentiations. These results suggest that fractional differentiation, demonstrates greater advantages in the analyzing of complex experimental designs.

**Figure 4:**
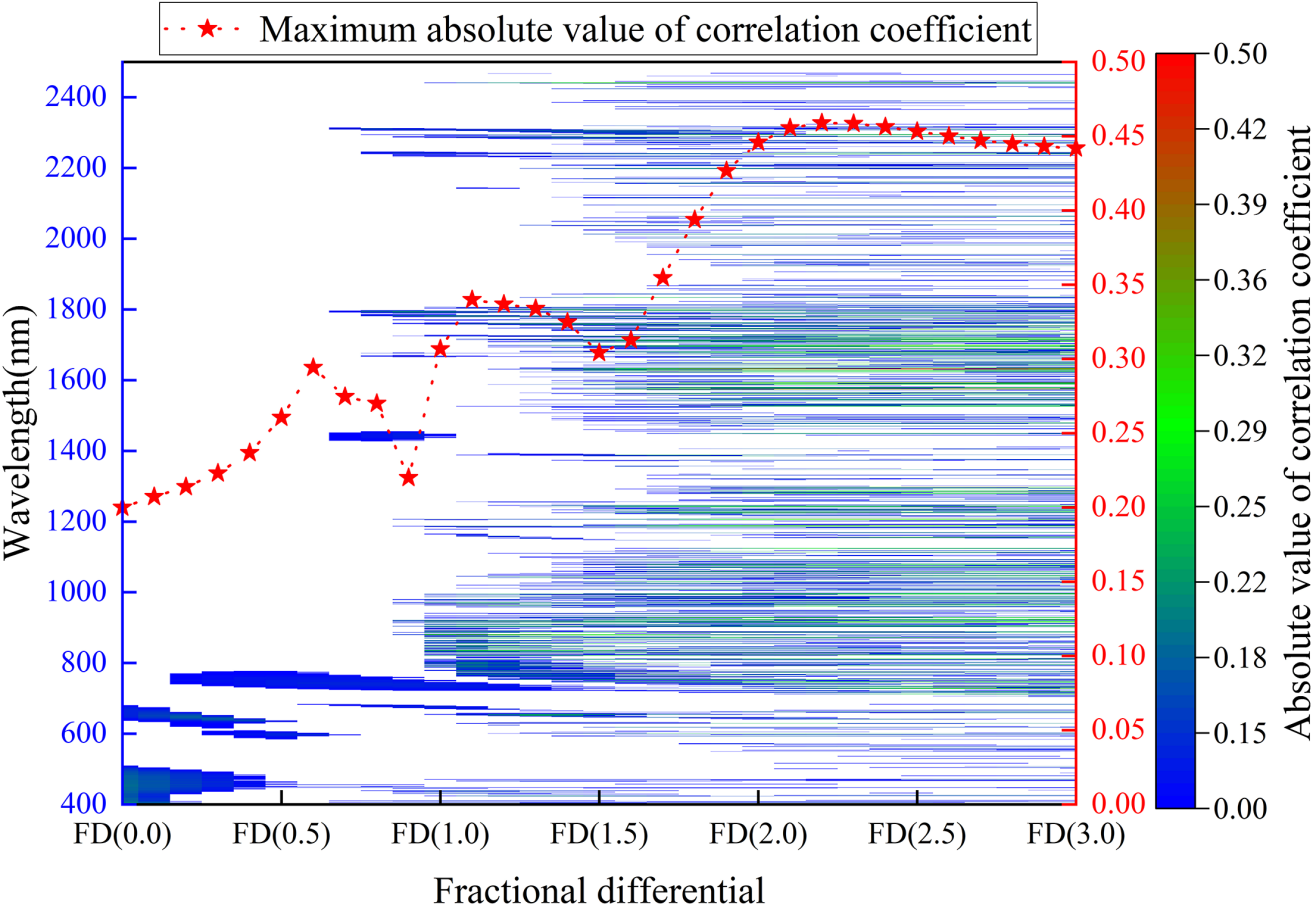
Absolute value distribution of correlation coefficients between fractional differentiation spectra and leaf potassium content, and the maximum absolute value of correlation coefficients for different fractional differentiations.

**Figure 4** illustrates the distribution of absolute correlation coefficients between fractional differentiation spectra and leaf potassium content across fractional differentiation orders ranging from FD (0.0) to FD (3.0), with wavelengths spanning from 400 to 2500 nm. Before fractional differentiation (FD (0.0)), the spectral bands between 400–505 nm and 640–680 nm show significant correlation with leaf potassium content, though the correlation coefficients are relatively low. As the order of fractional differential (FD) increases—particularly between FD (1.5) and FD (3.0)—the spectral information in the ranges of 700–1100 nm and 1400–1800 nm shows stronger correlations with leaf potassium content, with most correlation coefficients exceeding 0.2. The maximum absolute correlation coefficient generally increases from FD (0.0) to FD (2.2), reaching a peak value of 0.46, before declining at higher orders. These findings highlight that selecting an appropriate fractional differentiation order, such as FD (2.2), can effectively improve the correlation between spectral features and the target variable in practical applications.

### 3.3 Performance evaluation of individual models

**Figure 5:**
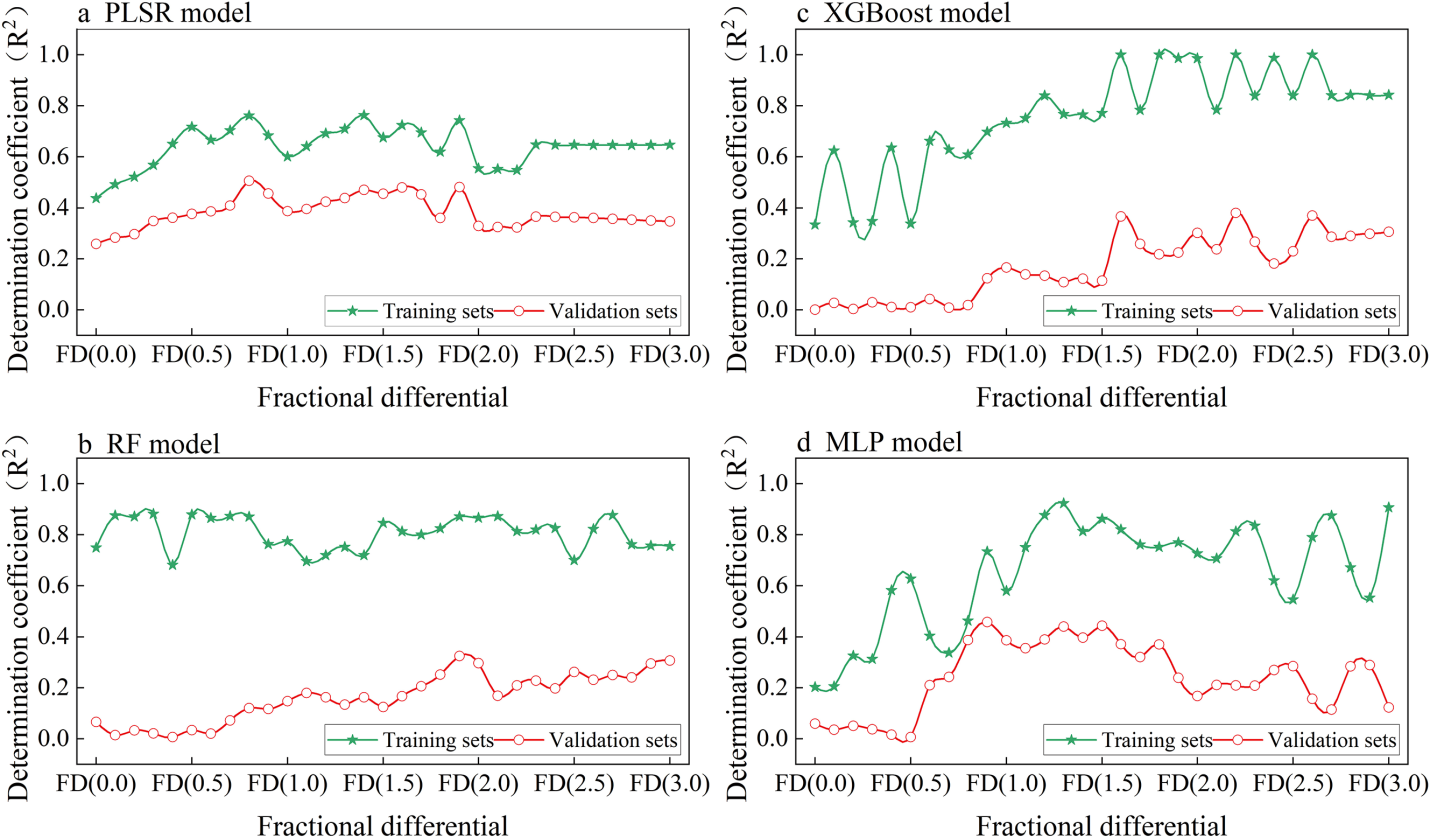
Determination Coefficients (R²) of different single models (**(a)** PLSR, **(b)** RF, **(c)** XGBoost, **(d)** MLP) for leaf potassium content estimation using fractional differentiation spectra: comparison of training and validation sets across different fractional differentiation orders (FD (0.0) to FD (3.0)).

The performance of the Partial Least Squares Regression (PLSR) model under fractional differentiation is shown in **Figure 5a**. Across the FD range from 0.0 to 3.0, the R² values for the training set consistently exceed those of the validation set by approximately 0.2 to 0.3, suggesting the presence of a certain level of overfitting in the PLSR model. The validation set achieves its highest R² value of 0.51 when the fractional differentiation is set to 0.8. Although the model’s fitting accuracy is relatively low, it demonstrates stable performance without significant overfitting.

As shown in **Figures 5b–d**, the RF, XGBoost, and MLP models all exhibit a marked discrepancy in R² values between the training and validation sets, reflecting a clear tendency toward overfitting. In comparison to RF and XGBoost, the MLP model demonstrates marginally superior validation performance, with a maximum R² of 0.46, outperforming RF (0.29) and XGBoost (0.38).

In summary, although the PLSR model has limited fitting accuracy in predicting leaf potassium content, it demonstrates good stability. The training set R² remains between 0.6 and 0.7, while the validation set R² stays between 0.3 and 0.5. In contrast, the RF, XGBoost, and MLP models perform well on the training set but poorly on the validation set, indicating potential overfitting. Therefore, among these four individual models, the PLSR model is the most suitable for estimating leaf potassium content.

### 3.4 Performance evaluation and analysis of combined models

**Figure 6:**
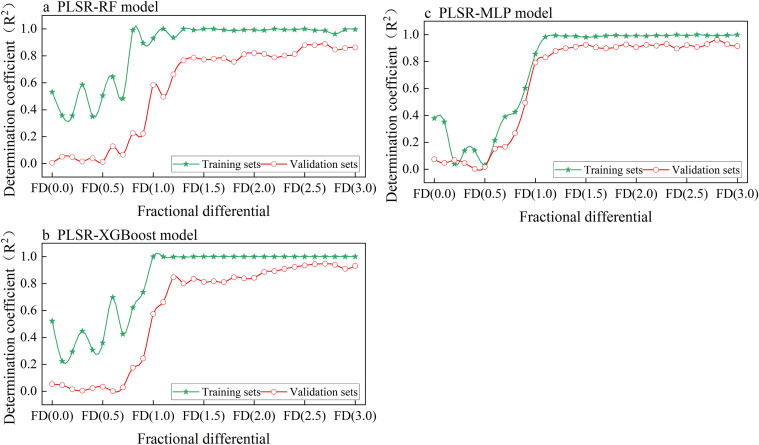
The relationship between different differential orders (FD) and the determination coefficient (R²) for training and validation sets across three models: **(a)** PLSR-RF, **(b)** PLSR-XGBoost, and **(c)** PLSR-MLP.

The PLSR-RF model (**Figure 6a**) demonstrates strong fitting and generalization capabilities, as evidenced by its stable performance across most FD settings. The training set achieves consistently high R^2^ values around 0.9, while the validation set maintains moderately high R² values ranging from approximately 0.75 to 0.89. Notably, within the FD range of 0.5 to 1.3, the validation performance improves sharply, with the R² value increasing from 0.01 to 0.77. The model achieves optimal performance at a fractional differentiation of FD (2.7), where the training set R² is 0.98, with MSE and MAE of 0.01 and 0.07, respectively. For the validation set, the R² value is 0.89, with MSE and MAE of 0.21 and 0.29, respectively.

The PLSR-XGBoost model shows significant fluctuations across different FD settings, particularly for the training set. Despite these fluctuations, the difference in R² values between the training and validation sets decreases significantly when the fractional differentiation exceeds 1.2 (**Figure 6b**). This indicates that the combined model effectively mitigates overfitting. When the fractional differentiation is set to FD (2.7), the model performance reaches its peak, with R², MSE, and MAE values are 0.99, 1.8*10^-5^, and 0.003 for the training set, and 0.94, 0.1, and 0.22 for the validation set, respectively. These findings indicate that PLSR combined with XGBoost provides more stable predictions under higher fractional differentiation levels.

The PLSR-MLP model performs poorly at low fractional differentiation values (FD < 0.8), with validation R² remaining below 0.4 between FD (0.2) and FD (0.6). Notably, at FD (0.3), the model exhibits signs of underfitting, as indicated by similarly low performance on both the training and validation sets. This suggests that the MLP has limited adaptability to raw data or data processed with low-order fractional differentiation (**Figure 6c**). However, as FD increases, the model’s performance improves significantly. At FD (2.8), the R² values for both the training and validation sets reach 0.99 and 0.96, respectively, with MSE and MAE values of 0.01 and 0.05 for the training set, and 0.07 and 0.16 for the validation set, indicating excellent model performance at this optimal order.

**Figure 7:**
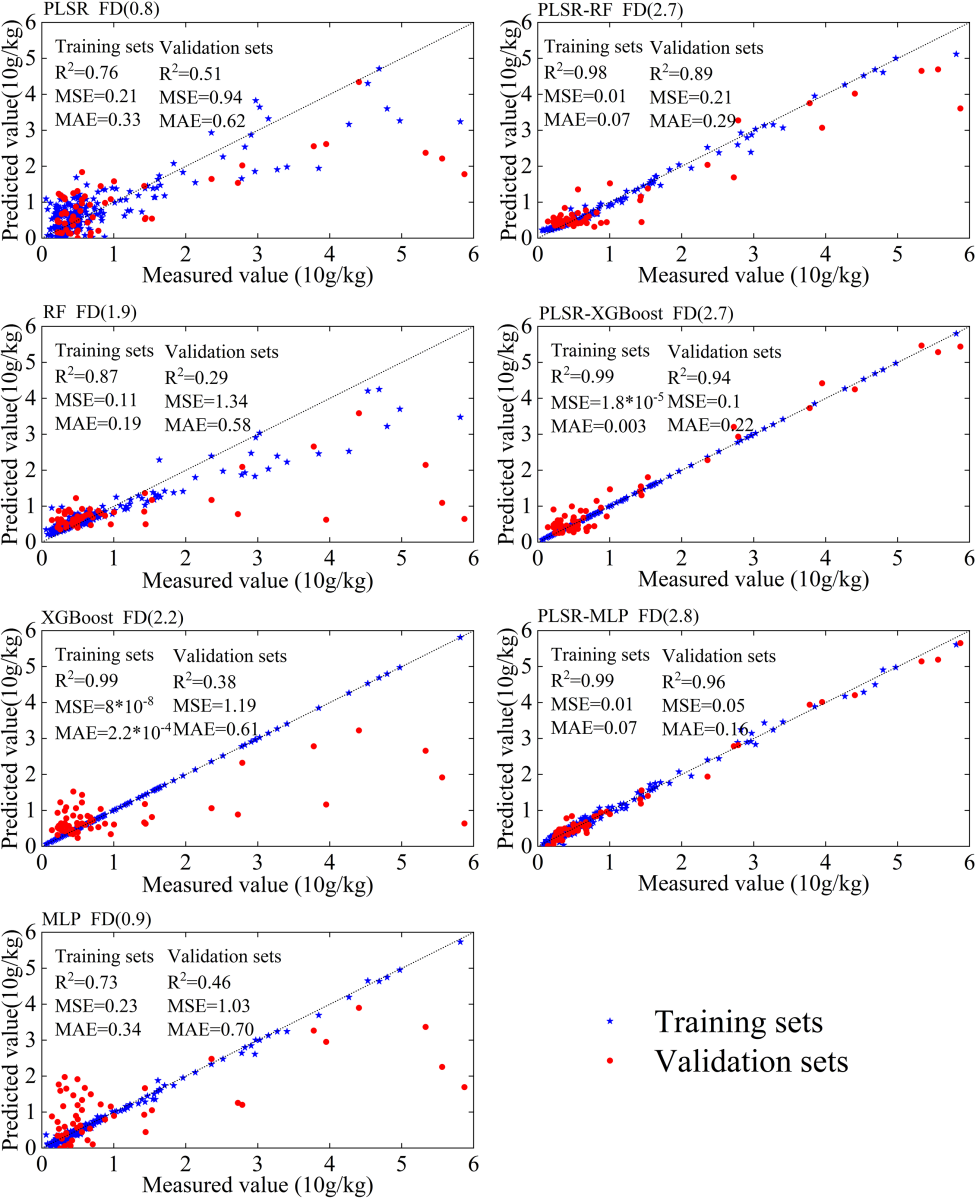
Prediction accuracy of leaf potassium content for each model at the optimal fractional differentiation order, showing the performance of individual models and combined models with evaluation metrics such as R², MSE, and MAE for both training and validation sets.

Overall, the three combined models exhibit distinct responses to fractional differentiation. PLSR-RF improves with increasing FD but shows signs of overfitting. PLSR-XGBoost generalizes well when FD > 1.0, despite early instability. While PLSR-MLP achieves the highest accuracy in this study (**Figure 7**), PLSR-XGBoost involves fewer hyperparameter adjustments, demonstrates high computational efficiency, and facilitates easy deployment Therefore, although PLSR-MLP is the optimal model in terms of predictive performance, PLSR-XGBoost may offer a more practical solution for real-world potassium prediction tasks, especially in scenarios with limited computational resources or where rapid deployment is required.

### 3.5 Model comparison and selection of the optimal model

In this study, seven models, namely PLSR, RF, XGBoost, MLP, PLSR-RF, PLSR-XGBoost, and PLSR-MLP, were applied to predict the plant leaf potassium content using spectral differentiation transformation techniques in the karst region of Guangxi Province. The optimal fractional differentiation prediction results for each model are shown in **Figure 7**. Based on the coefficient of determination (R²) on the validation sets, the top three models are PLSR-MLP (R²=0.96), PLSR-XGBoost (R²=0.94), and PLSR-RF (R²=0.89), respectively. In comparison, the RF model alone showed the worst performance, with an R² of only 0.29 on the validation sets.

Among these seven models, the PLSR-RF, PLSR-XGBoost, and PLSR-MLP models all effectively predict potassium content in plant leaves in the southwestern karst region. Relative to individual models, the three combined models exhibit improvements of 206%, 147%, and 108% in R^2^ on the validation set, respectively. These substantial gains suggest that the combined modeling approach effectively mitigates overfitting and enhances generalization capability.

### 3.6 Advantages of fractional differentiation

The fractional differentiation is determined to be the optimal spectral transformation approach for all seven models (**Table 1**). The application of fractional differentiation significantly enhances the models’ performance in estimating leaf potassium content. For the PLSR model, the optimal fractional differentiation is FD (0.8), resulting in a validation R² of 0.51, a marked improvement over the 0th order (R² = 0.26), 1st order (R² = 0.39), 2nd order (R² = 0.33), and 3rd order (R² = 0.35). The PLSR-RF model achieves its best performance at FD (2.7), with a validation R² of 0.89, significantly outperforming the 0th order (R² = 0.005), 1st order (R² = 0.58), 2nd order (R² = 0.82), and 3rd order (R² = 0.86).The PLSR-XGBoost model performs optimally at FD (2.7), with a validation R² of 0.94, significantly outperforming the 0th order (R² = 0.08), 1st order (R² = 0.58), 2nd order (R² = 0.83), and 3rd order (R² = 0.89). Finally, the PLSR-MLP model achieves its highest validation R² of 0.96 at FD (2.8), outperforming all integer orders from 0.0 to 3.0.

**Table 1:** Performance comparison of seven models at different fractional differentiation orders (0.0, 1.0, 2.0, 3.0) and the optimal fractional differentiation order, based on evaluation metrics including R², MSE, and MAE for both individual and combined models.

Model	Orders	Training Sets R2	Training Sets MSE	Training Sets MAE	Validation Sets R2	Validation Sets MSE	Validation Sets MAE
PLSR	FD (0.0)	0.44	0.49	0.49	0.26	1.41	0.77
	FD (1.0)	0.60	0.35	0.40	0.39	1.17	0.60
	FD (2.0)	0.55	0.39	0.40	0.33	1.28	0.60
	FD (3.0)	0.65	0.31	0.35	0.35	1.25	0.58
	**FD (0.8)**	**0.76**	**0.21**	**0.33**	**0.51**	**0.94**	**0.62**
RF	FD (0.0)	0.75	0.22	0.27	0.07	1.78	0.75
	FD (1.0)	0.77	0.2	0.25	0.15	1.63	0.71
	FD (2.0)	0.87	0.11	0.19	0.29	1.31	0.59
	FD (3.0)	0.76	0.21	0.25	0.3	1.43	0.64
	**FD (1.9)**	**0.87**	**0.11**	**0.19**	**0.29**	**1.34**	**0.58**
XGBoost	FD (0.0)	0.33	0.58	0.47	0.001	1.9	0.76
	FD (1.0)	0.72	0.23	0.31	0.17	1.59	0.69
	FD (2.0)	0.98	0.01	0.09	0.3	1.33	0.61
	FD (3.0)	0.84	0.14	0.21	0.31	1.32	0.61
	**FD (2.2)**	**0.99**	8*10^-8^	2.2*10^-4^	**0.38**	**1.19**	**0.61**
MLP	FD (0.0)	0.01	0.85	0.65	0.19	1.55	0.75
	FD (1.0)	0.42	0.51	0.42	0.45	1.05	0.7
	FD (2.0)	0.73	0.23	0.32	0.17	1.59	0.97
	FD (3.0)	0.91	0.08	0.17	0.12	1.67	0.98
	**FD (0.9)**	**0.73**	**0.23**	**0.37**	**0.46**	**1.03**	**0.7**
PLSR-RF	FD (0.0)	0.51	0.41	0.39	0.005	1.91	0.77
	FD (1.0)	0.93	0.06	0.15	0.58	0.79	0.49
	FD (2.0)	0.99	0.007	0.06	0.82	0.34	0.36
	FD (3.0)	0.99	0.004	0.03	0.86	0.26	0.32
	**FD (2.7)**	**0.98**	**0.01**	**0.07**	**0.89**	**0.21**	**0.29**
PLSR-XGBoost	FD (0.0)	0.52	0.41	0.38	0.05	2.0	0.77
	FD (1.0)	0.99	1.4*10^-6^	8.4*10^-4^	0.58	0.81	0.51
	FD (2.0)	0.99	3.1*10^-5^	0.004	0.84	0.3	0.33
	FD (3.0)	0.99	1.9*10^-5^	0.003	0.93	0.13	0.24
	**FD (2.7)**	**0.99**	1.8*^10-5^	**0.003**	**0.94**	**0.1**	**0.22**
PLSR-MLP	FD (0.0)	0.38	0.55	0.45	0.07	1.77	0.71
	FD (1.0)	0.86	0.12	0.21	0.79	0.39	0.35
	FD (2.0)	0.99	0.007	0.06	0.91	0.06	0.16
	FD (3.0)	0.99	0.001	0.02	0.91	0.04	0.19
	**FD (2.8)**	**0.99**	**0.01**	**0.07**	**0.96**	**0.05**	**0.16**

This table presents the R^2^, MSE, and MAE for the training and validation sets under the optimal fractional order, with the best-performing results shown in bold.

The results show that the optimal differentiation orders in all seven models are fractional rather than integer. This highlights the advantage of fractional differentiation in improving the accuracy and robustness of leaf potassium content estimation.

## 4 Discussion

### 4.1 Distribution of sensitive wavelengths

This study demonstrates that the spectral ranges of 700–1100 nm and 1400–1800 nm are critical for accurately estimating potassium content in plant leaves. Previous studies have identified the 964–1024 nm range as important for detecting potassium status in mature rubber tree leaves (Hu et al., 2024). In addition, specific wavelengths such as 720 nm and 1027 nm have been shown to play essential roles in predicting potassium content in rapeseed leaves (Zhang et al., 2013). The sensitive band in the 1400–1800 nm range identified in this study also aligns closely with the findings of Pimstein et al. (2011), further validating the relevance of this region for potassium estimation. Potassium is an essential ion in plant cells, involved in regulating osmotic pressure, activating enzymatic processes, and controlling stomatal dynamics (Nieves-Cordones et al., 2014; Yu et al., 2023). These physiological activities influence leaf cellular structure and water status, thereby indirectly affecting spectral reflectance. In the 700–1100 nm range, particularly within the near-infrared region (700–900 nm), spectral responses are strongly associated with internal leaf structure, which is sensitive to variations in tissue density and cellular arrangement. Since potassium plays a key role in water transport, cell turgor, and tissue development, changes in potassium levels can induce structural modifications that alter reflectance in this region (Lyu et al., 2023). Moreover, the short-wave near-infrared region (900–1100 nm) captures spectral signals related to leaf water content and biochemical composition, both of which are closely linked to potassium-mediated regulation (Dos Santos et al., 2023).

The presence of sensitive bands in the 1400–1800 nm range is closely linked to the various physiological roles of potassium in plant growth. Potassium influences leaf water transpiration by regulating stomatal opening, which in turn affects spectral reflectance (Lin et al., 2024). Consequently, potassium-sensitive bands are often found near the peak wavelengths of water absorption, such as 1450 nm and 1950 nm (Yu et al., 2023). However, some wavelengths farther from these water absorption peaks also show high sensitivity, likely due to changes in plant chemical composition and physiology under the unique environmental conditions of the karst regions. Previous studies have demonstrated significant differences in stoichiometric characteristics between plants in karst and non-karst regions (Zhang et al., 2019). Potassium is crucial for activating enzymes involved in starch, protein, and fat synthesis, as well as promoting the synthesis of plant hormones that regulate meristem growth (Amirruddin et al., 2020). These functions may contribute to the sensitive bands distanced from water absorption peaks. Therefore, the presence of such bands in the 1400–1800 nm range likely reflects potassium’s regulatory effects on physiological traits linked to long-term adaptation of plants to the karst environment.

### 4.2 The capabilities of fractional differentiation

Spectral data are often affected by instrument noise, environmental conditions, sample surface scattering, and background signals (Liu et al., 2023). Preprocessing techniques help mitigate these interferences, yielding a purer spectral signal that prevents the model from being affected by irrelevant signals and reduces errors (Li et al., 2025). Among these techniques, differentiation—particularly fractional differentiation—has emerged as a powerful method for capturing subtle spectral details and improving the accuracy of spectral-based estimations.

While traditional preprocessing techniques such as SNV and MSC effectively reduce scattering effects and smooth spectra, they are limited in handling high-noise spectral data (Oliveri et al., 2019). Differentiation processing of near-infrared spectra effectively removes noise while extracting subtle inflection points and spectral changes (Wang et al., 2018). Yang et al. (2022) demonstrated that applying differentiation to crop spectra significantly improves model prediction accuracy. Similarly, Shen et al. (2020) found that fractional differentiation significantly improves the accuracy of soil organic matter (SOM) content estimation. These studies highlight the significant advantages of differentiation in spectral preprocessing. Our findings similarly show that differentiation enhances the correlation between leaf potassium content and spectral reflectance, thereby improving estimation accuracy.

Differentiation includes both integer-order and fractional differentiation (Jin and Wang, 2022). Integer-order differentiation typically involves the first and second differentiations. However, the large intervals between these first and second differentiations result in significant differences between the nth and (n+1)th differentiation curves. This limitation causes integer-order differentiation to overlook finer spectral details (Anon, 2020). In contrast, fractional differentiation can extract detailed spectral information over smaller intervals while minimizing the introduction of excessive high-frequency noise (Zununjan et al., 2024; Song et al., 2023). The advantages of fractional differentiation stem from its unique mathematical structure, which, through the Grünwald-Letnikov definition, achieves a generalized difference structure, smooth attenuation, and long memory effects (Scherer et al., 2011). This enables fractional differentiation to more accurately capture spectral detail variations in data with complex background noise. Ge et al. (2022) demonstrated that fractional differentiation is highly effective for processing hyperspectral data in soil salinization risk assessment, with models using fractional differentiation proving more stable than those using integer-order differentiation. This conclusion from Ge et al. (2022) aligns with our findings, where fractional differentiation outperformed integer-order differentiation in estimating potassium content in plant leaves in the karst region.

However, the application of fractional differentiation also presents challenges. Low-order differentiation transformations provide limited improvement in correlation, while higher-order differentiation does not significantly enhance correlation coefficients between spectral reflectance and potassium content. Additionally, the optimal fractional differentiation varies across models, and similar studies on nutrient inversion in plant leaves suggest that the best fractional differentiation should be chosen based on the specific model being used.

### 4.3 Control overfitting

The results indicate that the RF, XGBoost, and MLP models generally exhibit overfitting (**Figure 5**). Due to their strong nonlinear fitting abilities (Bentéjac et al., 2021), these models tend to capture noise and irrelevant features when handling high-dimensional data, resulting in overfitting (Ying, 2019).

Common methods to control overfitting include dimensionality reduction, regularization, cross-validation, feature selection (Barbosa et al., 2024), and ensemble models. Several studies have explored the application of these methods in controlling overfitting. For example, Teresa et al. (2022) showed that dimensionality reduction effectively addresses over-parameterization in deep learning. Du et al. (2024) estimated rapeseed growth parameters using an ensemble learning algorithm, achieving better performance than individual machine learning models. For dimensionality reduction, we employed a PLS-based PCA method to extract latent variables that are highly correlated with the target variable. These latent variables were used as input features for the RF, XGBoost, and MLP models, effectively reducing the risk of overfitting in complex datasets.

In addition, hyperparameter optimization is a crucial strategy for mitigating overfitting and improving model generalization (Bischl et al., 2023). By tuning parameters such as the number of estimators, learning rate, and maximum tree depth (for RF and XGBoost), or the number of hidden layers and neurons (for MLP), models can better balance bias and variance. In this study, we employed grid search combined with cross-validation to optimize the key hyperparameters of each model, thereby reducing overfitting and enhancing predictive robustness. These findings are consistent with previous studies, which have demonstrated that well-tuned models generally outperform those using default configurations, particularly in high-dimensional datasets (Quan, 2024).

Combining dimensionality reduction with machine learning shows great potential for predicting nutrient content in plant leaves. For instance, Mahajan et al. (2024) used a PLSR-based machine learning model to predict potassium content in cashew leaves, achieving an R² of 0.66. Zhou et al. (2024) combined PCA with machine learning to predict cadmium content in lettuce leaves, obtaining an R² of 0.92 for the validation set. In our study, potassium content estimation in karst plants achieved an R² of 0.96 in the prediction set. This result confirms the effectiveness of PLS-based dimensionality reduction for retrieving leaf nutrient content across multiple species. This approach provides a valuable reference for future research.

In summary, combined machine learning models effectively control overfitting and enhance prediction performance. However, our research is limited to the leaf scale, and further validation is needed for their effectiveness in controlling overfitting when applied to UAV or satellite platforms. Future studies should explore the applicability of these models at larger scales and with higher-resolution data to comprehensively assess their generalization and practical value. Moreover, selecting the best model should not rely solely on prediction accuracy; factors such as model complexity, training time, and computational cost must also be taken into account to ensure the model’s feasibility and efficiency in real-world applications.

## 5 Conclusions

This study identifies key spectral bands (700–1100 nm, and 1400–1800 nm) that are critical for estimating potassium content in plant leaves. These bands correspond to important physiological processes, including photosynthesis, pigment concentration, and water regulation, which are influenced by potassium. Fractional differentiation effectively reduces noise and captures subtle spectral features, significantly improving the accuracy of potassium estimation compared to traditional integer-order differentiation.

Furthermore, the study addresses overfitting in machine learning models by combining dimensionality reduction, and advanced algorithms such as Random Forest (RF), Extreme Gradient Boosting (XGBoost), and Multilayer Perceptron (MLP). This integrated approach resulted in a high prediction accuracy (R² = 0.96) for potassium content in karst region plants.

In summary, this research advances potassium estimation through hyperspectral data by optimizing data preprocessing, and enhancing model performance. These findings provide valuable insights for plant nutrient monitoring, particularly in complex ecological environments, and offer a foundation for future research on large-scale remote sensing applications.

## Data Availability

The datasets presented in this article are not readily available because The data that has been used is confidential. Requests to access the datasets should be directed to Wen He, hw@gxib.cn.
